# Omission of tranexamic acid does not increase the amount of perioperative blood transfusions in patients undergoing one-level spinal fusion surgery: a retrospective propensity score-matched noninferiority study

**DOI:** 10.1007/s00402-022-04494-2

**Published:** 2022-06-16

**Authors:** Jonas Alfitian, Max Joseph Scheyerer, Axel Rohde, Volker Schick, Tobias Kammerer, Robert Schier

**Affiliations:** 1 University of Cologne, Faculty of Medicine and University Hospital Cologne, Department of Anaesthesiology and Intensive Care Medicine, Kerpener Str. 62, 50937 Cologne, Germany; 2 University of Cologne, Faculty of Medicine and University Hospital Cologne, Department of Orthopaedics and Trauma Surgery, Kerpener Str. 62, 50937 Cologne, Germany

**Keywords:** Tranexamic acid, Perioperative transfusions, Thromboembolism, Spine surgery

## Abstract

**Introduction:**

Application of tranexamic acid (TXA) in spine surgery is very frequent even without signs of hyperfibrinolysis, although its beneficial blood-saving effects are offset by harmful adverse events such as thromboembolic incidents. Thus, we investigated whether in relatively less invasive spinal procedures such as one-level posterior spinal fusion, omission of TXA affects the requirement for blood transfusions.

**Methods:**

We conducted a retrospective propensity score-matched noninferiority study with 212 patients who underwent one-level posterior spine fusion and who were stratified according to whether they received TXA intraoperatively at our tertiary care center. The primary endpoint was the volume of transfused packed red cells. Testing for noninferiority or equivalence was performed by two one-sided testing procedure (TOST) with a priori defined noninferiority margins ($\delta$).

**Results:**

After propensity score matching a total of five patients (11.6%) treated with TXA were transfused compared with five patients (11.6%) who did not receive TXA. The majority of patients (51.2%) had a risk-increasing condition. The risk difference (no TXA–TXA) of intraoperative transfusion was − 4.7% (CI 90% − 13.62 to 4.32%), and omitting TXA was noninferior ($\delta$ = $\pm$  10%). The mean intergroup difference in transfused volume (no TXA–TXA) was − 23.26 ml intraoperatively (CI 90% − 69.34 to 22.83 ml) and − 46.51 ml overall (CI 90% − 181.12 to 88.1 ml), respectively, suggesting equivalence of TXA omission ($\delta$ = $\pm$ 300 ml). The hemoglobin decline between both groups was also equivalent (with $\delta$ = $\pm$ 1 g/dl) both on the first postoperative day ($\Delta \Delta$ Hb = 0.02 g/dl, CI 90% − 0.53 to 0.56 g/dl) and at discharge ($\Delta \Delta$ Hb = − 0.29 g/dl, CI 90% − 0.89 to 0.31 g/dl).

**Conclusion:**

We demonstrated that requirement of transfusion is rare among one-level fusion surgery and the omission of TXA is noninferior with regard to blood transfusion in high-risk patients undergoing this procedure. Therefore, the prophylactic use of TXA cannot be recommended here, suggesting to focus on alternative blood conservation strategies, if necessary.

## Introduction

In recent years, surgical procedures of the spine benefit from perioperative blood conservation strategies including autologous blood donation, the utility of intraoperative cell salvage (cell saver), reduction of central venous pressure and the use of synthetic antifibrinolytic medications [1, 2]. Tranexamic acid (TXA) is the most frequently used antifibrinolytic agent and in the past 10 years, and numerous studies investigated the risks and benefits of such pharmacological blood conservation strategies [3–5].

In 1960s, TXA was introduced to clinical practice with several studies in cardiac and noncardiac surgery showing a reduction of blood transfusions [6]. A large national claims data analysis with 82.512 patients undergoing shoulder arthroplasty revealed a marked decrease in the transfusion risk with no increases in the complication risk [7]. The use of TXA in total hip arthroplasty was analysed in an evidence review including numerous RCTs and meta-analyses finding TXA administration to be able to significantly reduce blood loss [8–10]. Negative effects were an increased risk of seizures at higher doses and the authors recommend cautious use of TXA in patients with renal dysfunction, hypercoagulable states, hypersensitivity to TXA, coronary or vascular stent placement, thromboembolic disease, or cerebrovascular event within prior 6 months [11–13]. However, they identified a pitfall in the existing body of evidence concerning the harmful effects of TXA in high-risk patient groups. Alarmingly, many studies excluded these high-risk patients (i.e. risk for venous thromboembolism or pulmonary embolism) and, therefore, the efficacy and safety of TXA in this cohort is uncertain. Generally, the Clinical Practice Guidelines emphasize on the use of TXA in total joint arthroplasty [8, 10].

The use of TXA in spine surgery has been discussed controversial, as systematic reviews and meta-analyses of the available literature have emphasized the need for higher powered studies with more consistency in terms of the procedure, dosage, and regimen utilized [4, 14, 15]. Although, a growing body of evidence of clinical trials demonstrates that the use of TXA in spine surgery can lead to significant reduction of perioperative blood loss and transfusion, still an area of continued research exists to confirm that the increased potential of thrombogenesis outweighs the clinical benefits of TXA [16]. As a matter of importance, in practice, TXA is also used fairly often in relatively low-bleeding spinal procedures such as dorsal one-level fusion procedures, although the occurrence of hyperfibrinolysis and thus a rationale for TXA is very rare for this type of surgery. To add evidence to the current controversy about perioperative use of TXA in one-level fusion surgery including high-risk patients, we conducted a retrospective propensity score-matched noninferiority study in a tertiary care center. Primary endpoint was the difference of transfusion volume of packed red blood cells. Secondary endpoints were difference of hemoglobin level, length of stay (LOS), length of ICU stay (LOICUS), and the incidence of thromboembolic complications.

## Materials and methods

### Data sources and study population

We conducted a retrospective, propensity score-matched, noninferiority study in a tertiary care center including patients who had undergone elective one-level fusion surgery. Surgery types included posterior instrumentation and fusion, posterior lumbar interbody fusion (PLIF) and transforaminal lumbar interbody fusion (TLIF). The underlying pathologies included degenerative disc disease and spondylolisthesis. Patients were identified within a 5-year period (January 2015–January 2020) within a prospectively maintained hospital database. Exclusion criteria were only age under 18 years or pregnancy, allowing to obtain a representative sample of the patient population undergoing this surgery type. All patients received perioperative care according to an established *Enhanced Recovery after Surgery* (ERAS) pathway. Preoperative risk stratification was performed within the anesthesiological premedication consult with medical history taking, medication with anticoagulants or antiplatelet drugs, physical examination, along with routine laboratory parameter testing. Patients with either coronary artery disease, peripheral arterial disease, active malignant tumour disease, a history of thrombosis, or were ASA 3 or higher were classified as high risk, as they were at increased risk of a complicative outcome due to increased blood loss on one hand and an increased risk of thromboembolic complications due to TXA on the other. Patients with limiting instable disease were not approved for elective surgery.

### Collection of patient data

Data were extracted from the medical records by two investigators and cross-referenced. Hemoglobin levels were obtained the day before surgery. Estimated blood loss (EBL), volume of transfused packed red blood cells, and administration of TXA were documented during surgery. EBL was determined by the volume of blood aspirated minus the irrigation fluid. In rare cases where large quantities of gauze compresses were soaked with blood, the EBL was corrected according to the individual estimate by the anesthesiologist. Finally, hemoglobin levels were collected both on the first postoperative day and the day of discharge. Moreover, the volume of transfused packed red cells throughout the remaining hospital stay was ascertained. Postoperative complications were assessed for the incidence of thromboembolic events. Specifically, these were classified as deep vein thrombosis, pulmonary embolism, myocardial infarction, and ischemic stroke. Data were also collected on the length of intensive care unit stay and the overall hospital stay.

### Study design

212 patients were enrolled in our study. Since the chance of TXA administration during surgery was driven by multiple determinants, we performed propensity score matching to ensure all patients after matching were equally likely to receive TXA. The decision on TXA administration was made on an individual basis for each patient at the discretion of the conducting anesthesiologist. Apart from the present hemorrhage situation, the patient’s medical history, medication as well as intraoperative hemodynamic and metabolic changes were taken into account. Consequently, to account for potential bias, EBL, age, ASA status, history of thrombosis or pulmonary embolism, known coronary artery disease, peripheral arterial disease, anticoagulant intake, and baseline hemoglobin level were implemented as clinically important determinants in a logistic regression model for odds of receiving TXA. The standardized mean difference was calculated for each covariate to demonstrate adequate balance after matching if lower than 0.1. After propensity score matching, 86 patients were remaining.

The volume of transfused red blood cell concentrates was the primary endpoint of our study. Secondary endpoints included the probability of transfusion, the difference in hemoglobin value from baseline on the first postoperative day and on the day of discharge [$\Delta$Hb(d1) and $\Delta$Hb(discharge)], and the length of hospital stay (LOS) and intensive care unit stay (LOICUS). Data were evaluated for noninferiority of abstaining from TXA. We also examined whether TXA affects the risk of postoperative thromboembolic events.

### Statistical analysis

Statistical analysis was performed using the R Language version 4.0.3 [17]. Continuous variables are reported as mean with standard deviation (SD). Categorial data are represented as count (n) with frequency (%).

Testing for noninferiority or equivalence was performed by two one-sided testing procedure (TOST) [18]. The 90% confidence interval was calculated to achieve a 0.05 significance level after two-sided testing. A two-sided noninferiority margin $\delta$ was set a priori for all analyses. In cases where lower values are the favoured outcome, noninferiority was assumed when the 90% confidence interval excluded $+\delta$. Equivalence was inferred when the 90% confidence interval additionally excluded $-\delta$. For the volume of transfused packed red blood cells, $\delta$ was set as 300 ml, which is roughly corresponding to the volume of a single unit of packed red blood cells. Furthermore, the risk of being transfused was calculated for both groups and the risk difference was then calculated, with $\delta$ set to 10%. For $\Delta$Hb, we defined 1 g/dl as $\delta$. For LOICUS and LOS, $\delta$ was specified as 1 day and 3 days, respectively.

Multivariable logistic regression modelling was used for estimation of the impact of TXA administration on the risk of thromboembolic events. Venous and arterial thromboembolic events were assessed separately in view of their different pathophysiological mechanisms. The models were adjusted to common risk factors such as age, sex, history of thrombosis, known hypercoagulability, coronary heart disease, tumour disease and peripheral arterial disease.

A two-tailed *P* value of less than 0.05 was considered statistically significant. All statistical analyses and their interpretation were independently reviewed by a qualified statistician.

## Results

### Study population

212 patients undergoing elective spine surgery were enrolled in our study. 78 (36.8%) received at least 1 g of TXA during surgery. The patient population comprised high-risk patients with a total of 82 (38.7%) ASA 3 and 4 (1.9%) ASA 4 patients, respectively. Since the chance of receiving TXA was primarily driven by intraoperative blood loss alongside multiple factors, we performed a propensity score matching approach to address any confounding that was likely to be present. Mean estimated blood loss was significantly higher in patients receiving TXA than in those who did not receive TXA (Mean $\pm$ SD: 971 $\pm$ 620 ml vs. 517 $\pm$ 258 ml, *P* $=$ 5.34e-11). Following propensity score matching, there were 86 patients remaining, of whom 43 (50%) received TXA and 43 (50%) did not. After matching, estimated blood loss, representing a strong confounder on the primary endpoint, was not different in both groups (Mean $\pm$ SD: 657 $\pm$ 270 ml vs. 637 $\pm$ 314 ml, *P* $=$ 0.758). However, after matching, the proportion of high-risk patients with 39 (45.3%) ASA 3 and 1 (1.2%) ASA 4 patients, respectively, was similar to the non-matched population. 44 (51.2%) patients in the matched cohort had either coronary artery disease, peripheral arterial disease, active malignant tumour disease, a history of thrombosis, or were ASA 3 or higher and were thus considered high-risk patients. Patient characteristics are demonstrated in Table 1. Furthermore, no difference was found between both groups regarding age, sex, existing tumour disease, coronary artery disease or peripheral arterial disease, hypercoagulability, intake of anticoagulant or antiplatelet drugs, or estimated blood loss. All subsequent analyses were performed among the matched study population.

**Table 1:** Characteristics of the study population after propensity score matching

Variable	Statistic	Overall, *N* = 86	Tranexamic acid		*p* value^a^
			No TXA	1 g TXA	
Age (years)	Mean (SD)	68 (14)	69 (12)	67 (17)	0.5
Sex					> 0.9
Female	*n* (%)	38 (44%)	19 (44%)	19 (44%)	
Male	*n* (%)	48 (56%)	24 (56%)	24 (56%)	
ASA status					0.6
1	*n* (%)	6 (7.0%)	2 (4.7%)	4 (9.3%)	
2	*n* (%)	40 (47%)	19 (44%)	21 (49%)	
3	*n* (%)	39 (45%)	21 (49%)	18 (42%)	
4	*n* (%)	1 (1.2%)	1 (2.3%)	0 (0%)	
Coronary artery disease	*n* (%)	9 (10%)	5 (12%)	4 (9.3%)	> 0.9
Peripheral arterial disease	*n* (%)	5 (5.8%)	3 (7.0%)	2 (4.7%)	> 0.9
History of thrombosis	*n* (%)	16 (19%)	8 (19%)	8 (19%)	> 0.9
Tumour disease	*n* (%)	6 (7.0%)	4 (9.3%)	2 (4.7%)	0.7
Hypercoagulability	*n* (%)	4 (4.7%)	2 (4.7%)	2 (4.7%)	> 0.9
Acetylsalicylic acid intake	*n* (%)	18 (21%)	10 (23%)	8 (19%)	0.6
Heparin intake	*n* (%)	11 (13%)	6 (14%)	5 (12%)	0.7
Other antiplatelet drug intake	*n* (%)	4 (4.7%)	4 (9.3%)	0 (0%)	0.12
Surgery duration (mins)	Mean (SD)	156 (49)	152 (49)	161 (49)	0.4
Hemoglobin before surgery (g/dl)	Mean (SD)	12.75 (2.23)	12.72 (2.49)	12.79 (1.96)	0.9
Estimated blood los (ml)	Mean (SD)	647 (292)	637 (314)	657 (270)	0.5
Hematokrit after surgery (%)	Mean (SD)	36.4 (6.7)	37.3 (7.1)	35.5 (6.2)	0.2

^a^Welch two sample *t* test; Pearson’s Chi-squared test; Fisher’s exact test; Wilcoxon rank sum test

### Blood transfusion

A total of four patients (9.3%, Unmatched cohort: 11 (14.1%)) treated with TXA were transfused intraoperatively compared with two patients (4.7%, Unmatched cohort: 3 (2.2%)) who did not receive TXA. Accordingly, four patients (9.3%, Unmatched cohort: 12 (15.4%)) patients in the TXA group were transfused in the postoperative period versus four patients (9.3%, Unmatched cohort: 7 (5.2%)) patients in the no-TXA group. Transfusion of fresh frozen plasma or platelets was not necessary in any case. All outcome variables are shown in Table 2.

**Table 2:** Outcome parameters of the matched study population

Variable	Statistic	Overall, *N* = 86	Tranexamic acid	
			No TXA	1 g TXA
Intraoperative transfusion	*n* (%)	6 (7.0%)	2 (4.7%)	4 (9.3%)
Postoperative transfusion	*n* (%)	8 (9.3%)	4 (9.3%)	4 (9.3%)
Intraoperative transfusion volume (ml)	Mean (SD)	35 (128)	23 (107)	47 (147)
Postoperative transfusion volume (ml)	Mean (SD)	81 (275)	70 (234)	93 (314)
Total transfusion volume (ml)	Mean (SD)	116 (373)	93 (294)	140 (441)
Hemoglobin day one after surgery (g/dl)	Mean (SD)	9.86 (1.78)	9.82 (2.01)	9.90 (1.53)
Hemoglobin discharge day (g/dl)	Mean (SD)	10.17 (1.61)	10.29 (1.57)	10.06 (1.65)
Length of stay (days)	Mean (SD)	18 (12)	17 (10)	19 (14)
Length of ICU stay (days)	Mean (SD)	0.44 (1.86)	0.58 (2.46)	0.30 (0.94)
Postoperative venous thrombosis	*n* (%)	0 (0%)	0 (0%)	0 (0%)
Postoperative pulmonary embolism	*n* (%)	1 (1.2%)	0 (0%)	1 (2.3%)
Postoperative myocardial infarction	*n* (%)	1 (1.2%)	0 (0%)	1 (2.3%)
Postoperative stroke	*n* (%)	1 (1.2%)	1 (2.3%)	0 (0%)

**Fig. 1:**
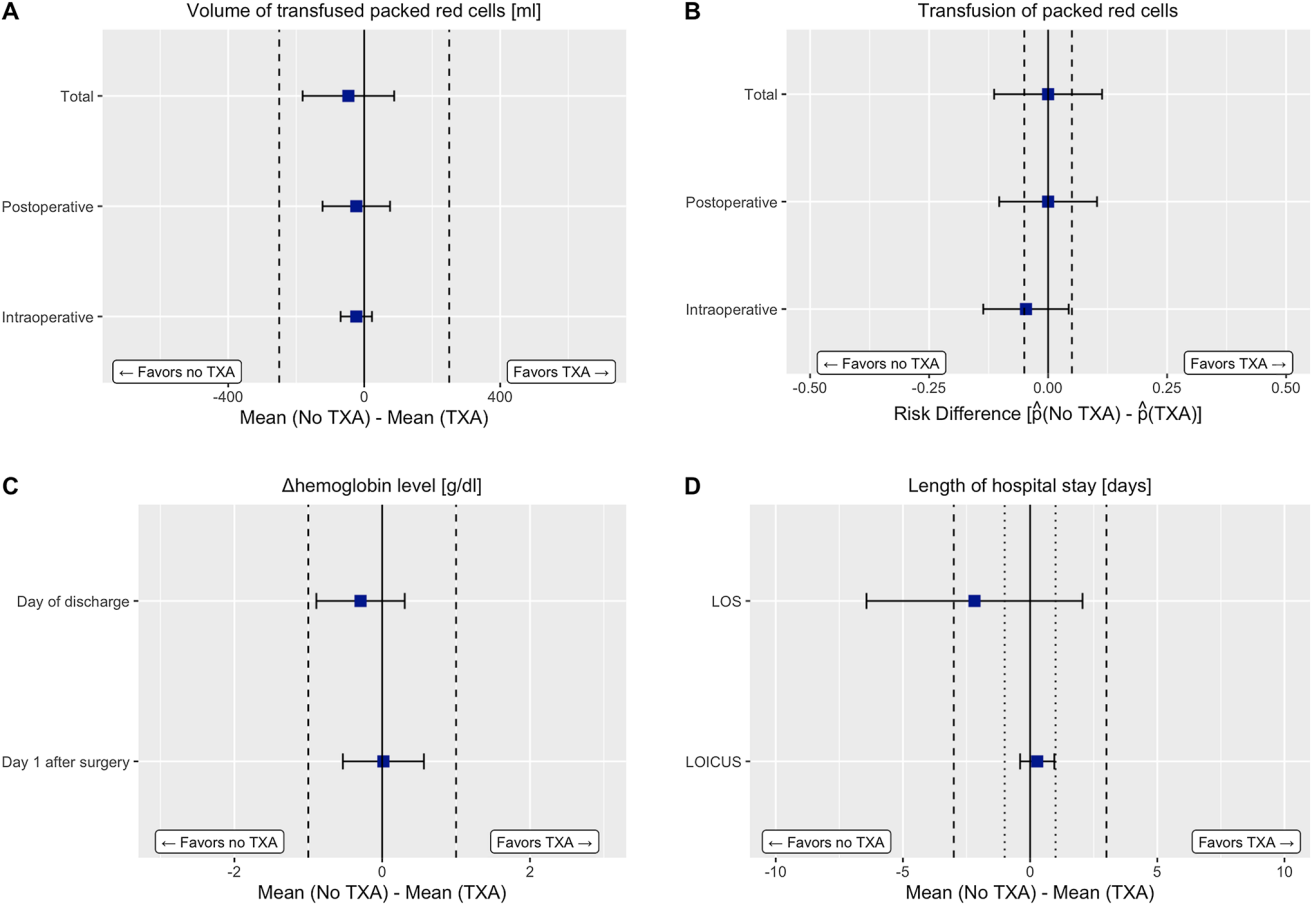
Results of two one-sided testing for noninferiority. Dashed lines represent the prespecified noninferiority and equivalence margins. In D the dotted lines depict the margins for LOICUS, and the dashed lines for LOS

Figure 1A demonstrates that TOST revealed equivalence of omitting TXA in terms of the primary endpoint of intraoperative ($\Delta$Transfusion Volume = -23.26 ml, CI 90% − 69.34 to 22.83 ml), postoperative ($\Delta$Transfusion Volume = − 23.26 ml, CI 90% − 122.5 to 75.99 ml) and total ($\Delta$Transfusion Volume = − 46.51 ml, CI 90% − 181.12 to 88.1 ml) transfusion volume with predefined noninferiority margin falling outside the confidence interval.

However, noninferiority for the risk of transfusion as a secondary endpoint could only be inferred for the intraoperative period ($\Delta \widehat{p}$= − 4.7%, CI 90% − 13.62 to 4.32%). Although omitting TXA had an almost zero risk difference both for the postoperative course ($\Delta \widehat{p}$= 0%) and total stay ($\Delta \widehat{p}$= 0%), equivalence could not be inferred due to inconclusiveness of the TOST with the confidence intervals including $\delta$ (Fig. 1B).

In the matched cohort, no difference in preoperative hemoglobin levels could be verified (Mean $\pm$ SD: TXA group: 12.8 $\pm$ 2 g/dl, No TXA group: 12.7 $\pm$ 2.5 g/dl, *P* = 0.893). On the first postoperative day, those patients who did not receive TXA had a mean decrease of hemoglobin value of 2.9 $\pm$ 1.6 g/dl, while this was 2.9 $\pm$ 1.4 g/dl in the control group. Consequently, $\Delta \Delta$b(d1) was 0.02 g/dl and TOST demonstrated that the confidence interval (CI 90% − 0.53 to 0.56 g/dl) excluded − $\delta$ and + $\delta$ with $\delta$ = 1 g/dl, thus making omission of TXA equivalent to treatment with TXA (Fig. 1C). Similarly, on the discharge day, the decline in hemoglobin $\Delta$Hb(discharge) was found equal in both groups (Mean $\pm$ SD: TXA group: 2.7 $\pm$ 1.5 g/dl, No TXA group: 2.4 $\pm$ 1.8 g/dl), indicating equivalence of omitting TXA according to TOST ($\Delta \Delta$Hb(discharge) =  − 0.29 g/dl, CI 90% − 0.89 to 0.31 g/dl).

### Length of hospital stay

Mean overall length of hospital stay was 19.4 $\pm$ 13.5 days in the control group and 17.3 $\pm$ 9.8 days in the no-TXA group, and considering $\delta$ = 3 days, omitting TXA was noninferior (TOST: $\Delta$ LOS = − 2.2 days, CI 90% − 6.4 to 2.1 days) as demonstrated in Fig. 1D. Additionally, both groups were equivalent respective to the length of ICU stay (TOST: $\Delta$ LOICUS = 0.3 days, CI 90% − 0.4 to 1 days).

### Thromboembolic complications

Perioperative thromboembolic complications were categorised as venous thromboembolism comprising deep vein thrombosis and pulmonary embolism, and arterial thromboembolism which included myocardial infarction and ischemic stroke. The overall incidence of these complications was very low, involving 0 patients (0%, Unmatched cohort: 1 (0.5%)) suffering deep vein thrombosis, 1 (1.2%, Unmatched cohort: 2 (0.9%)) with pulmonary artery embolism, 1 (1.2%, Unmatched cohort: 1 (0.5%)) with myocardial infarction and 1 (1.2%, Unmatched cohort: 1 (0.5%)) with ischemic stroke. Multivariable logistic regression failed to confirm a significant role of TXA as a predictor of thromboembolic events (Table 3). A significant role as a predictor could only be identified for deep vein thrombosis or pulmonary embolism among the covariates age and sex, with male sex decreasing the risk (OR − 553; 95%CI − 57,669 to 12,857; *P* = 0.006) and rising age reducing the risk (OR − 17; 95%CI − 1,674 to 515; *P* = 0.007). Moreover, no relationship was found between TXA and myocardial infarction or stroke (Table 3). However, taking into account the very low incidence of thromboembolic events in the study cohort, it is important to interpret these results with considerable caution.

**Table 3:** Results of multivariable logistic regression model for estimation of predictors for the occurence of thromboembolic complications

Predictor	Venous thrombosis or pulmonary embolism			Stroke or myocardial infarction		
	OR^a^	95% CI	*P* value	OR^a^	95% CI	*p* value
TXA administered	56	− 3,244, 3,653	0.054	− 0.40	− 4.0, 3.0	0.8
Age (years)	− 17	− 1,674, 515	0.007	0.11	− 0.03, 0.42	0.2
Sex			0.006			0.054
Female	–	–		—	—	
Male	− 553	− 57,669, 12,857		19	− 703, NA	
Tumor disease	101	− 12,382, 12,584	> 0.9			
History of thrombosis	319	− 9,190, 9,828	> 0.9			
Hypercoagulability	204	− 17,347, 17,756	> 0.9	− 15		0.9
Coronary heart disease				− 18		0.4
Peripheral arterial disease				− 18		0.6

*OR*  odds ratio, *CI* confidence interval

^a^Multivariable logistic regression

## Discussion

In recent years, a key goal of “patient blood management” programs has been to reduce perioperative blood loss. Increased need for blood transfusion is accompanied with higher morbidity and mortality in a number of medical fields [19–21]. Therefore, testing the efficacy of blood-saving strategies like inhibition of fibrinolysis using protease inhibitors, such as TXA, has been subject of research for many years. Still, controversy exists whether to use TXA as prophylaxis in spine surgery as benefits (reducing blood loss) and harmful side effects (i.e. venous thromboembolism) must be outweighed in these patients, especially in those with underlying risk factors [2]. Furthermore, application should be critically reviewed for commonly low-bleeding procedures such as dorsal one-level fusions, where hyperfibrinolysis is unlikely to occur.

In our tertiary care center, we found that the use of TXA in patients undergoing one-level fusion spine surgery did not decrease the perioperative need for transfusion. We observed that omitting TXA (no-TXA group) was equivalent to the use of TXA (TXA group) in terms of total volume of transfused red blood cells in these patients. Hemoglobin decline remained equivalent between the TXA and no-TXA group both on first postoperative day ($\Delta$Hb(d1)) and upon discharge ($\Delta$Hb(discharge)). Moreover, LOS and LOICUS did not differ between the groups. As perioperative thromboembolic events in relation to the use of TXA play a significant role in clinical practice, we analysed the incidence of deep venous thrombosis, pulmonary embolism, stroke and myocardial infarction. However, given the very low overall incidence, no reliable statement can be made about the relative risk of thromboembolic events associated with TXA. Yet, TXA should be used with caution, given prior exclusion of high-risk patients with known coronary artery disease, cerebrovascular insufficiency or vascular or coronary stent implantation, or a thromboembolic event history in the preceding 6 months in those prospective studies [22, 23]. Thus, the safety and efficacy of this drug remains uncertain within this relevant patient cohort. In our retrospective analysis, these high-risk patients were not excluded up front and the decision whether to give or omit TXA was made on an individual basis, thus reflecting the representative patients for this surgery type and the practice for decision of TXA application. The existing randomized controlled trials that tested the efficacy and safety of TXA in spine surgery were analysed in two key meta-analyses. Cheriyan et al. found 11 RCTs with a total of 644 patients and reported a reduction of intraoperative, postoperative, and total blood loss as well as a reduced number of blood transfusions in association with TXA [4]. In contrast to these findings, Yuan et al. performed a meta-analysis including 685 patients showing that TXA was not able to decrease the incidence of blood transfusions in patients undergoing scoliosis surgery, even though the total blood loss was reduced in patients receiving TXA [24]. These results were confirmed by a multicentre, randomised, placebo-controlled trial with 95 patients undergoing major spine surgery, where TXA did not significantly reduce transfusion requirements, but significantly reduced perioperative blood loss [25]. These studies still offer some uncertainty about the blood transfusion-saving effect of TXA. Therefore, to add to this debate, our findings from a tertiary center suggest that abstaining from TXA in high-risk patients has no impact on the necessity or quantity of packed red blood cells being transfused. Remarkably, overall blood loss and transfusion incidence was much higher in these studies, which does not translate to our patient population. The incidence of transfusion was very low in our unmatched patient cohort with 25 patients (11.8%) of the overall cohort before matching, which is, however, to be expected for this type of surgery. Yet, conversely, TXA was used far more often in 78 patients (36.8%), which is consistent with the practice of potentially unjustified intraoperative TXA administration among this surgery type. Indeed, we were able to demonstrate that this discrepancy between frequency of TXA administration and frequency of transfusions was not due to an underlying effect of TXA.

Yet, there are still some limitations inherent in our study. Whilst a retrospective study design poses a potential weakness, we were able to obtain a representative sample of the corresponding patient population and have methodically offset the drawbacks of a retrospective design. Still, a prospective study in the future would clearly be favoured. TXA administration was not decided according to predefined criteria but on an individual basis, although this was addressed by propensity score matching. Furthermore, our method for determining EBL does not allow for a millilitre-precise measurement. However, EBL was only relevant as one of several parameters to determine the individual chance of receiving TXA for propensity score matching, and not as an endpoint to investigate a potential blood-saving effect of TXA. Without data on the amount of crystalloid infusion solution administered intraoperatively, it is possible that the hemoglobin value on the first postoperative day may be distorted by dilution due to massive fluid administration, which may have occurred in individual cases. Since the incidence of thromboembolic complications was extremely low overall, usage may be assumed to be safe, but a reliable statement on the relative risk of TXA use is not feasible.

### Conclusion

Based on our data, we can conclude that TXA can be omitted in high-risk patients undergoing one-level spine fusion surgery without affecting the need for transfusion. The overall transfusion requirement is minor for this type of procedure. Furthermore, refraining from TXA was noninferior or equivalent regarding decline in hemoglobin level and both LOICUS and LOS. However, no reliable statement can be made about the safety of TXA usage with regard to the relative risk for thromboembolic complications.

## Data Availability

The datasets generated during and/or analysed during the current study are available from the corresponding author on reasonable request.
